# A prenatally diagnosed case of Meckel–Gruber syndrome with novel compound heterozygous pathogenic variants in the *TXNDC15* gene

**DOI:** 10.1002/mgg3.614

**Published:** 2019-03-09

**Authors:** Konstantin Ridnõi, Marek Šois, Eve Vaidla, Sander Pajusalu, Larissa Kelder, Tiia Reimand, Katrin Õunap

**Affiliations:** ^1^ Centre for Perinatal Care Women's Clinic, East‐Tallinn Central Hospital Tallinn Estonia; ^2^ Department of Clinical Genetics Institute of Clinical Medicine, University of Tartu Tartu Estonia; ^3^ Fetal Ultrasound Screening Centre Tallinn Estonia; ^4^ United Laboratories Department of Clinical Genetics Tartu University Hospital Tartu Estonia; ^5^ Department of Genetics Yale University School of Medicine New Haven Connecticut; ^6^ Centre of Pathology Diagnostic Clinic, East‐Tallinn Central Hospital Tallinn Estonia; ^7^ Department of Biomedicine Institute of Biomedicine and Translational Medicine, University of Tartu Tartu Estonia

**Keywords:** ciliopathies, fetal ultrasound, Meckel–Gruber syndrome, prenatal, *TXNDC15* gene

## Abstract

**Background:**

Meckel–Gruber syndrome (MKS) is a well‐known rare disease that can be detected on prenatal ultrasound. Meckel–Gruber syndrome has very heterogeneous etiology; at least, 17 genes have been described in association with MKS. The characteristic findings in fetuses affected by MKS are encephalocele (usually occipital), postaxial polydactyly, and polycystic dysplastic kidneys. However, the association of the *TXNDC15* gene with MKS has been reported only once before in three consanguineous families.

**Methods:**

We report a new case of MKS diagnosed at 12 + 1 weeks of gestation with typical ultrasound findings, but with novel compound heterozygous pathogenic variants in the *TXNDC15* gene identified by whole‐exome sequencing (WES).

**Results:**

This is the second clinical report supporting *TXNDC15* as a novel causative gene of MKS, and the first describing a case in a non‐consanguineous family with causative compound heterozygous mutations.

**Conclusions:**

Meckel–Gruber syndrome is a very heterogeneous syndrome in terms of the associated causal genes. In the first‐line diagnosis, we used an next‐generation sequencing (NGS)‐based large gene panel, but only 10 MKS genes were available on the platform used. In the case of prenatal ultrasound findings that are highly suggestive of MKS and a negative NGS MKS gene panel, WES should also be performed to not miss rare gene associations.

## 1 INTRODUCTION

Meckel–Gruber syndrome (MKS, OMIM#249000) is a rare, lethal autosomal recessive ciliopathy caused by mutations in one of at least 17 associated genes (Hartill, Szymanska, Sharif, Wheway, & Johnson, 2017). Its incidence has been estimated at one in 135,000 livebirths worldwide (Auber et al., 2007). A recent European study reported that the mean prevalence of MKS is 2.6 per 100,000 births (Barisic et al., 2015), but it can be much higher in certain populations, for example, in Finland, where MKS incidence is one in 9,000 livebirths (Salonen, Norio, Opitz, & Reynolds, 1984), with *MKS1* gene mutations originally identified in the Finnish population (Kyttala et al., 2006). The incidence of MKS can be even higher in endogamous populations with high consanguinity rates. In Qatar, MKS incidence is one in 2,000 livebirths (Al‐Belushi et al., 2016). Meckel–Gruber syndrome presents with central nervous system malformations, usually occipital encephalocele, and postaxial polydactyly, polycystic kidneys, and malformation of the liver ductal plate. Prenatal or perinatal death occurs in 100% of cases (Knopp et al., 2015). The *TXNDC15 *gene has been associated with MKS in only one previous clinical report, where homozygous loss‐of‐function variants were detected in three consanguineous families (Shaheen et al., 2016). However, recently published CRISPR (clustered regularly interspaced short palindromic repeats)‐based screen results strongly suggest that *TXNDC15* indeed is a novel ciliopathy gene (Breslow et al., 2018).

Here, we report a prenatal case of MKS caused by compound heterozygous variants in the *TXNDC15* gene.

## 2 CLINICAL REPORT

A 33‐year‐old Estonian patient was referred for clinical geneticist consultation at 13 + 0 weeks after abnormal ultrasound investigation results on the first‐trimester scan. She was otherwise healthy, but had one ectopic pregnancy in the past. Her partner was a 33‐year‐old healthy Estonian male. The parents were not known to be related. The family history was negative for severe genetic disorders.

**Figure 1:**
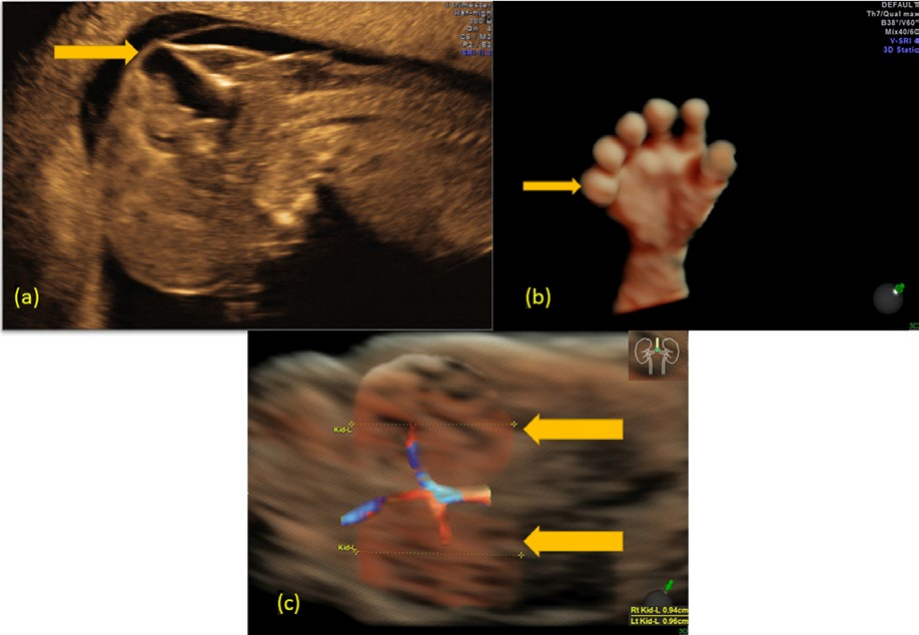
(a) Occipital encephalocele in two‐dimensional high‐resolution transvaginal ultrasound: midsagittal view shows the large occipital bone defect through which the meninges and cerebral parenchyma have migrated. (b) Postaxial polydactyly of one hand in three‐dimensional (3D) high‐resolution transvaginal ultrasound in surface mode demonstrating postaxial view of the extra digit (six digits). (c) Cystic renal dysplasia in 3D high‐resolution transvaginal ultrasound in constructed glass body mode: coronal view shows enlarged hyperechoic kidneys leading to distention of the abdomen

**Figure 2:**
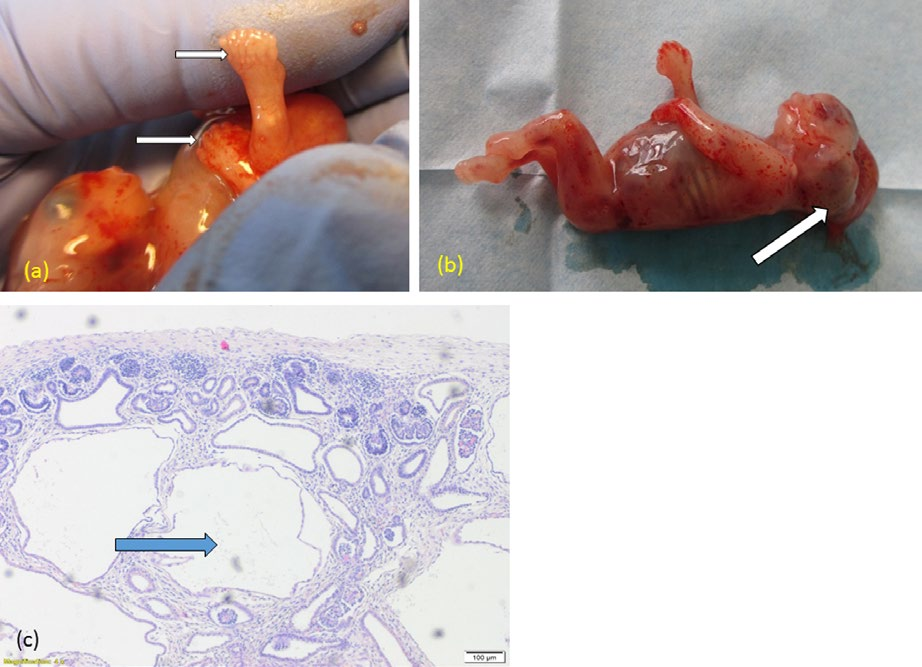
(a) Fetus after termination of pregnancy. Postaxial polydactyly is seen in the six fingers and seven toes (arrows). (b) Occipital encephalocele (arrow). (c) Histological findings in fetal kidneys: thin intermittent cortex of the kidneys with dysplastic cystic structures of varying sizes are lined with a single layer of immature cubic nephrogenic epithelium

Ultrasound examination revealed numerous anomalies: enlarged nuchal translucency (4.1 mm), bilateral polycystic kidneys, occipital encephalocele, and postaxial polydactyly on the hands and feet (Figure 1). Based on the ultrasound examination, there was a strong suspicion for MKS. After multidisciplinary counselling, the patient decided to terminate the pregnancy with post‐mortem autopsy. The autopsy showed occipital encephalocele (0.9 × 1.0 cm), bilateral enlarged polycystic kidneys (1.4 × 0.7 × 0.5 cm) with total kidney tissue mass of 0.503 g (normal at 12 weeks: 0.16 ± 0.04 g [Enid Gilbert‐Barness, Oligny, & Siebert, 2007]). Bilateral polydactyly of the hands (six fingers) and feet (seven toes) was also noted. No other anomalies were found. Histological study confirmed polycystic dysplastic kidneys (Figure 2).

## 3 METHODS

The Research Ethics Committee of the University of Tartu approved this study; protocol 263/M‐19 (date Oct 17, 2016).

First, next‐generation sequencing (NGS) was performed using an Illumina TruSight One sequencing panel. No pathogenic variants were found in the *MKS1*, *TMEM216*, *TMEM67*, *CEP290*, *RPGRIP1L*, *CC2D2A*, *NPHP3*, *TCTN2*, *B9D1*, and *B9D2* genes, which are known genes associated with MKS. Second, parents–offspring trio whole‐exome sequencing (WES) was performed. We identified two compound heterozygous mutations in the *TXNDC15 *gene: NM_024715.3:c.211dup p.(Gln71Profs*32) rs780024847 (inherited from the father) and NM_024715.3:c.635T>C p.(Leu212Pro) rs760579409 (inherited from the mother). Sanger sequencing confirmed these variants, which have never been described before in association with genetic disorders. According to the gnomAD database, such allele variants are very rare in the general population, with allele frequencies of 0.0029% and 0.00041%, respectively (Lek et al., 2016). The frameshift mutation c.211dup has been classified as a pathogenic variant due to its truncating effect on protein synthesis. The missense mutation c.635T>C has been classified as likely pathogenic due to it being very rare, in trans with the frameshift variant, and damaging predictions from multiple in silico prediction algorithms (SIFT, PolyPhen, CADD). Based on these findings, we concluded that in our case, two pathogenic variants in the *TXNDC15 *gene are likely to be the cause for the congenital malformations and MKS diagnosis. The family recurrence risk for affected offspring is 25%.

## 4 DISCUSSION

Homozygous mutations in the *TXNDC15 *gene causing MKS have been reported in one publication, where MKS was described in three consanguineous families: two Saudi and one Pakistani (Shaheen et al., 2016). The prenatal findings in one stillbirth were typical for MKS: polydactyly, enlarged cystic kidneys, and occipital encephalocele. In the first Saudi family, a homozygous NM_024715.3:c.672_686del:p.(Ser225_His229del) pathogenic variant was identified. The second Saudi family had several affected pregnancies with typical MKS findings, and in their case, a homozygous NM_024715.3:c.103+1G>A variant was present. The third family in the report was of Pakistani origin and had two children with MKS; the pathogenic homozygous variant NM_024715.3:c.956dupT,(p.Ser321Lysfs*15) was identified in that case (Shaheen et al., 2016). Here, we report the same prenatal findings: bilateral postaxial polydactyly, occipital encephalocele, and bilateral polycystic kidneys caused by compound heterozygous variants in the *TXNDC15 *gene. Recent experimental findings using a CRISPR‐based screen for ciliary disorders suggest that the *TXDNC15* gene, which encodes a thioredoxin domain‐containing transmembrane protein, is indeed a novel MKS gene (Breslow et al., 2018). In our case, we have identified frameshift mutation NM_024715.3:c.211dup p.(Gln71Profs*32), which causes reading frame change and results in formation of premature stop codon, which likely initiates nonsense‐mediated decay eliminating mRNA and altering the protein synthesis in this frame. In missense mutation NM_024715.3:c.635T>Cp.(Leu212Pro)rs760579409, which is located in thioredoxin domain, we see two possibilities: damaging the protein function, with possible residual activity or generation of new splice‐site exon in sequence which also can alter protein synthesis in this frame.

Meckel–Gruber syndrome is a very heterogeneous syndrome in terms of the associated causal genes. In the first‐line diagnosis, we used an NGS‐based large gene panel, but only 10 MKS genes were available on the platform used. In the case of prenatal ultrasound findings that are highly suggestive of MKS and a negative NGS MKS gene panel, WES should also be performed to not miss rare gene associations.

Meckel–Gruber syndrome is a lethal disorder. In Europe, 88% of prenatally detected cases are terminated during pregnancy (Barisic et al., 2015). The prenatal features of MKS, that is postaxial polydactyly, encephalocele, and polycystic kidneys, are usually profound and can be readily detected in the first trimester (Sepulveda, Sebire, Souka, Snijders, & Nicolaides, 1997). The findings of a large population‐based review estimating the incidence of the typical symptoms were as follows: encephalocele, 83.8%; polydactyly, 87.3%; and cystic kidney disease, 97.7% (Barisic et al., 2015). Therefore, targeted prenatal diagnosis of MKS is usually triggered by these findings. However, the presence of encephalocele is not specific for MKS. Only about 21% of fetuses with prenatally diagnosed encephalocele would have MKS (Weichert et al., 2017), and the same applies to the finding of polycystic kidney. The majority of confirmed hereditary cystic kidney disease found prenatally is autosomal recessive polycystic kidney disease (ARPKD), which is diagnosed in 81% of cases. Meckel–Gruber syndrome has been found in only 8% of such cases (Erger, Bruchle, Gembruch, & Zerres, 2017). Compared to ARPKD, cystic changes in the kidneys in MKS are seen much earlier in the pregnancy (Erger et al., 2017).

In summary, MKS is relatively frequent at least in some populations (Al‐Belushi et al., 2016; Salonen et al., 1984) and is lethal in all prenatal or perinatal cases (Barisic et al., 2015); therefore, attention should be focused on MKS in the case of early abnormal ultrasound findings such as postaxial polydactyly, encephalocele, and polycystic kidneys. However, some rare gene variants, including *TXNDC15 *gene variants, might be missed if these genes are not covered by routine gene panel NGS. Precise molecular diagnosis is important for further genetic counselling and the estimation of recurrence risk.

## CONFLICT OF INTEREST

None declared.
